# Case Report: Fatal *Pneumocystis jirovecii* Infection in an Elderly Man Receiving Adjuvant Paclitaxel and Trastuzumab Therapy for HER2-Positive Breast Cancer

**DOI:** 10.1155/crom/5515318

**Published:** 2025-05-13

**Authors:** Colin Vercueil, Hamza Ouaz, Emilie Schultz, Jean Marc Limacher

**Affiliations:** ^1^Oncology Department, Hopitaux Civils de Colmar, Colmar, France; ^2^Department of Supportive Care, Léon Bérard Cancer Center, Lyon, France

**Keywords:** benefit risk assessment, case reports, male breast cancer, opportunistic infection, pneumocystis infection

## Abstract

**Background:** *Pneumocystis jirovecii* pneumonia (PJP) is a well-recognized opportunistic infection in immunocompromised patients, particularly those with hematological malignancies or HIV infection. However, its occurrence in patients with solid tumors undergoing chemotherapy remains poorly characterized.

**Case Presentation:** We report the case of an 84-year-old male patient with HER2-positive breast cancer who developed severe PJP following adjuvant chemotherapy with paclitaxel and trastuzumab. The patient had no known immunosuppressive conditions and did not present chemotherapy-induced lymphopenia prior to the onset of symptoms. He was admitted with fever and dyspnea, 9 days after discontinuation of chemotherapy due to Grade 3 asthenia. Chest computed tomography (CT) revealed diffuse ground-glass opacities, and bronchoalveolar lavage confirmed the presence of *Pneumocystis jirovecii* DNA by PCR. Despite prompt initiation of sulfamethoxazole/trimethoprim and corticosteroids, the patient developed refractory acute respiratory distress syndrome (ARDS) and died after ICU admission.

**Discussion:** This case highlights the potential risk of PJP in elderly patients receiving adjuvant chemotherapy, even in the absence of evident immunosuppression. Given the increasing use of chemotherapy in older populations, a thorough risk–benefit assessment should be considered, especially when the expected survival benefit is limited. Current guidelines do not recommend systematic PJP prophylaxis in patients with solid tumors, yet emerging evidence suggests that chemotherapy-related lymphopenia may increase susceptibility to opportunistic infections.

**Conclusion:** Clinicians should maintain a high index of suspicion for opportunistic infections such as PJP in elderly patients undergoing chemotherapy, regardless of their immune status. This case underscores the importance of individualized risk stratification and vigilant monitoring to prevent and manage life-threatening complications.

## 1. Introduction

Pneumocystis is a common opportunistic infection in patients with severe T-cell immunodeficiency [1]. Occurrence of *Pneumocystis jirovecii* pneumonia (PJP) in patients with solid tumors is rare, half compared to those with hematological malignancies, yet comprehensive identification of risk factors in this population remains lacking [2].

Male breast cancer (MBC) is a rare disease and makes up only approximately 1% of all breast cancer [3]. Human epidermal growth factor receptor-2 (HER-2) overexpression in MBC occurs at a rate twice that observed in female breast cancer, with a prevalence of 40% compared to 19%.

We present the case of a male patient who developed PJP after the 9th course of paclitaxel, administered as part of adjuvant chemotherapy for HER2-positive breast cancer.

## 2. Case Presentation

### 2.1. Case History and Examination

The patient, an 84-year-old male, has no significant medical history. He underwent a mastectomy for a 2-cm hormone receptor-positive, HER2-positive, node-negative invasive ductal carcinoma. Based on the patient's favorable health condition as indicated by a Balducci I classification during the oncogeriatric assessment, adjuvant chemotherapy was recommended at the multidisciplinary team meeting. The patient was tested negative for human immunodeficiency virus (HIV) serology before starting chemotherapy. The patient had no known history of chronic pulmonary disease or preexisting respiratory comorbidities. Following current recommendations, treatment combining paclitaxel (reduced to 60 mg/m^2^ to minimize toxicity) and trastuzumab (loading dose of 4 mg/kg and subsequent doses 2 mg/kg) was administered according to the “Tolaney regimen” [4]. Premedication before each paclitaxel infusion included methylprednisolone 40 mg IV, as per institutional protocol. Treatment began 6 weeks after mastectomy. Treatment was discontinued after the 9th course due to Grade 3 asthenia. There was no lymphopenia observed in the blood count at this stage.

**Figure 1:**
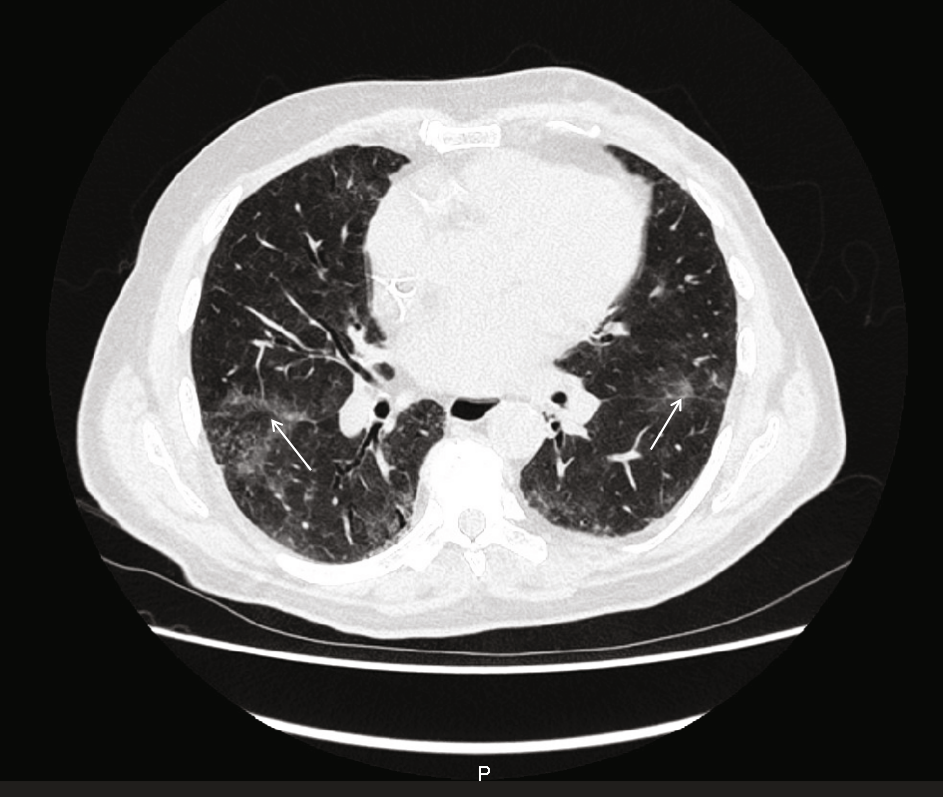
Coronal CT scan view illustrating bilateral ground-glass opacities indicative of diffuse interstitial pneumopathy. The scan was performed 9 days post the last chemotherapy administration.

The patient was admitted to the emergency department with fever and dyspnea, 9 days after the last administration of chemotherapy. Auscultation revealed diffuse crackling rales. Oxygen saturation was 70% on room air. A computed tomography (CT-scan) revealed diffuse ground-glass opacities (Figure 1), and blood analysis showed a decreased lymphocyte count of 400 cells/mm^3^ (normal range: 1000–4000 cells/mm^3^). CD4+ T cell count was not assessed, in line with our institutional practice for non-HIV patients.

### 2.2. Methods

The same day, bronchoscopy identified alveolar filling with foamy exudate. Bronchoalveolar lavage was performed in a segment of the right middle lobe. PCR for *Pneumocystis jirovecii* was positive in the bronchoalveolar lavage fluid. No fungal staining or culture was performed, as PCR positivity was considered sufficient for diagnosis. *β*-D-glucan testing was not performed, as this assay is not routinely available in our center. Treatment with intravenous sulfamethoxazole/trimethoprim (960 mg/4800 mg per day) and methylprednisolone (60 mg per day) was initiated immediately. The patient's condition necessitated admission to the intensive care unit (ICU) and orotracheal intubation, 2 days after his admission to the emergency department (i.e., on Day 11 postchemotherapy).

### 2.3. Conclusion and Results

Despite comprehensive interventions, including controlled assisted ventilation with 100% fraction of inspired oxygen (FiO_2_), the patient's clinical status progressively deteriorated, in link with a refractory acute respiratory distress syndrome (ARDS). On ICU Day 5 (i.e., Day 15 postchemotherapy), a consensus decision was made to limit therapeutic interventions. The patient passed away the following day.

## 3. Discussion

To our knowledge, this represents the first documented case of pneumocystis pneumonia in a male patient following adjuvant chemotherapy for early-stage breast cancer.

Notably, case reports have documented instances of PJP in breast cancer patients undergoing chemotherapy. A case of fatal pneumocystis was reported in Canada in 2012 in a female patient undergoing first-line paclitaxel and trastuzumab treatment for bone metastatic breast cancer [5]. The PJP diagnosis occurred after eight cycles of chemotherapy over 7 weeks when the lymphocyte count was at its lowest, measuring 400 cells/mm^3^. Susceptibility was attributed to factors such as the use of dexamethasone and lymphocyte suppression.

Interestingly, both infections occurred approximately at the same time (during the third month after treatment initiation). However, unlike our patient, the woman presented chemotherapy-induced lymphopenia. This highlights the risk of pneumocystis infection during chemotherapy, even without evident lymphopenia during chemotherapy.

Similarly, cases of PJP have been observed in breast cancer patients undergoing docetaxel, particularly among elderly or frail individuals, emphasizing the necessity for thorough monitoring of adverse effects with the expanding use of chemotherapy in this population [6].

Recognized risk factors for pneumocystis, aside from HIV infection, include prolonged use of corticosteroids (defined as > 20 mg prednisone equivalent daily for > 4 weeks), low CD4 cell counts, coexisting pulmonary diseases, and recent chemotherapy. In addition, although corticosteroid premedication with paclitaxel is standard practice to prevent hypersensitivity reactions, repeated weekly administration of methylprednisolone may also contribute to increased susceptibility to PJP, particularly in frail patients [7, 8]. Furthermore, having a solid tumor acts independently as a risk factor for increased mortality related to PJP [1].

Following the observation of two cases of HIV-negative pneumocystis in patients undergoing adjuvant chemotherapy for breast cancer, Tolaney et al. initiated a prospective cohort study [9]. Their investigation revealed chemotherapy-induced lymphopenia during neoadjuvant or adjuvant breast cancer treatment as a significant concern, notably around 5^th^ cycle, associated with an elevated susceptibility to opportunistic infections.

Current guidelines do not recommend systematic pneumocystis prophylaxis during treatment for solid tumors, except for temozolomide chemotherapy and prolonged use of corticosteroids [10].

This underscores the significance of implementing personalized medical strategies, as exemplified in our case study, where factors extending beyond the administration of paclitaxel contribute to the risk of *Pneumocystis jirovecii* infection.

Otherwise, the effectiveness of adjuvant chemotherapy in elderly individuals remains a subject of debate, prompting critical consideration of its benefits and risks in this specific age group.

The APT (adjuvant paclitaxel and trastuzumab) trial published in 2015 included 10% of patients aged over 70 years in their cohort [4]. Notably, within this cohort, adverse events of Grade 4 were observed in only 3 out of 406 patients, with no reported toxic deaths or severe infections.

A recent French propensity score-based study underscores the feasibility of chemotherapy in elderly early breast cancer patients, highlighting its potential for enhancing the overall survival and emphasizing the importance of avoiding undertreatment based on age [11].

However, when utilizing the PREDICT tool, an online prediction tool validated for use in elderly patients, the benefit appears debatable [12, 13]. Incorporating our patient and tumor profiles into this tool suggests that chemotherapy would offer only marginal benefit. Specifically, 68 out of 100 patients treated with hormone therapy and trastuzumab would survive 5 years, compared to 69 out of 100 patients treated with chemotherapy, trastuzumab, and hormone therapy, resulting in an increment of just one additional survivor due to breast cancer-related causes.

While adjuvant and neoadjuvant chemotherapies can reduce breast cancer mortality, there is acknowledgment that they may also increase mortality from other causes [14]. Making an ideal treatment choice requires a rigorous analysis that considers the trade-offs between benefits and potential risks, encompassing the broader context of patient health and the evolving landscape of medical progress.

In conclusion, we report a case of fatal PJP in an elderly male patient receiving adjuvant paclitaxel and trastuzumab therapy for HER2-positive breast cancer. This case emphasizes the importance of carefully assessing the benefits and risks of adjuvant chemotherapy in frail patients, where the potential benefits may be limited, and underscores the necessity of vigilant monitoring to prevent potential opportunistic infections.

## Data Availability

The data that support the findings of this study are available from the corresponding author upon reasonable request.
